# Targeted Delivery of Lidocaine in Breast Cancer Cells via Zeolitic Imidazolate Framework‐8 Nanoparticles

**DOI:** 10.1002/cphc.202401128

**Published:** 2025-06-24

**Authors:** Nicola di Nicola, Luca Giacchi, Marco Vandone, Marcello Crucianelli, Leonardo Guidoni, Valentina Colombo, Nadia Rucci, Andrea Lazzarini

**Affiliations:** ^1^ Department of Physical and Chemical Sciences (DSFC) University of L’Aquila Via Vetoio (“A.C. De Meis” building) 67100 L’Aquila Italy; ^2^ UdR INSTM of L’Aquila University of L’Aquila Via Vetoio (“A.C. De Meis” building) 67100 L’Aquila Italy; ^3^ Department of Biotechnological and Applied Clinical Sciences (DISCAB) University of L’Aquila Via Vetoio (“A.C. De Meis” building) 67100 L’Aquila Italy; ^4^ Department of Chemistry and UdR INSTM of Milano University of Milano Via C. Golgi 19 67100 Milano Italy

**Keywords:** anticancer application, lidocaine, pH‐regulated delivery, targeted delivery, ZIF‐8 nanoparticles

## Abstract

The aim of this article is to use zeolitic imidazolate framework‐8 (ZIF‐8) metal–organic frameworks (MOFs) as drug delivery system for lidocaine, a local anesthetic with recently discovered antitumoral effects, in particular for the early‐stage treatment of breast cancer. A previously optimized ZIF‐8 synthetic method is employed, and the prepared material is deeply characterized with X‐ray powder diffraction (XRPD), surface area analysis, scanning electron microscopy (SEM), and thermogravimetric analysis (TGA). After confirming the successful synthetic outcomes, a tailored loading procedure is developed to incorporate lidocaine within the ZIF‐8 pores. The effectiveness of this loading process is verified using UV–Vis spectroscopy and TGA, while SEM and attenuated total reflectance‐mid infrared spectroscopy further confirms the presence of lidocaine within the MOF pores. Afterwards, the pH‐responsive drug release by dialyzing the lidocaine@ZIF‐8 sample is tested either at physiological or at weakly acid pH values by UV spectroscopy on the dialysis solutions. Finally, the MDA‐MB‐231 breast cancer cells viability of lidocaine@ZIF‐8 system is tested and compared with the free drug and the MOF alone, confirming its significant capabilities in the reduction of cells viability.

## Graphical abstract

Drug repositioning of lidocaine from local anesthetic to theranostic agent for breast cancer cells is enhanced by its encapsulation inside ZIF‐8 metal–organic framework.

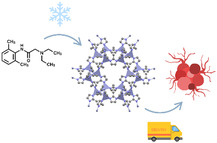

## 1 Introduction

In recent years, metal–organic frameworks (MOFs) have attracted increasing attention due to their remarkable combination of structural versatility, high surface area, and porosity,^[^ ^1^ ^]^ making them suitable for a wide range of applications,^[^ ^2^ ^]^ including gas storage,^[^ ^3^ ^]^ catalysis,^[^ ^4^ ^]^ and biomedical uses.^[^ ^5^ ^]^ Among these, zeolitic imidazolate framework‐8 (ZIF‐8) has emerged as a standout MOF, known for its exceptional chemical and thermal stability,^[^ ^6^ ^]^ as well as its excellent biocompatibility.^[^ ^7^ ^]^ ZIF‐8 consists of imidazolate ligands coordinated with zinc ions, forming a highly tunable framework with a large surface area and a porous structure, ideal for selective adsorption and encapsulation,^[^ ^8^ ^]^ either of pollutants molecules,^[^ ^9^ ^]^ or of pharmacological active molecules.^[^ ^10^ ^]^ Considering the last application, ZIF‐8 holds significant potential in drug delivery systems,^[^ ^11^ ^]^ among other biomedical applications.^[^ ^12^ ^]^ Its porous structure allows efficient encapsulation of drug molecules, while its chemical stability helps to prevent premature drug degradation. Additionally, ZIF‐8 is pH‐sensitive, enabling stimuli‐responsive drug release.^[^ ^13^ ^]^ This feature is particularly advantageous for targeted therapies, as it minimizes systemic side effects and allows for the precise targeting of specific environments, such as the acidic microenvironment of tumor cells and inflamed areas in general.^[^ ^14^ ^]^ Breast cancer is the most common malignant neoplasm among women worldwide, and is treatable in ≈70%–80% of patients if recognized at an early‐stage, nonmetastatic disease.^[^ ^15^ ^]^ However, advanced breast cancer with metastasis to distant organs is considered untreatable with the currently available therapies.^[^ ^16^ ^]^ Treatment strategies vary depending on the molecular subtype of the cancer. Future therapeutic approaches in breast cancer aim at the individualization of treatment, with a focus on de‐escalation and escalation of therapy based on tumor biology and early response to treatment.^[^ ^17^ ^]^ In addition to ongoing therapeutic innovations, ensuring equitable access to these advancements globally remains a critical challenge in the future of breast cancer care. Several retrospective studies have suggested a potential impact of anesthesia on cancer patient survival.^[^ ^18^ ^]^ In particular, regional anesthesia has been associated with a reduced risk of cancer recurrence, with one proposed explanation being the opioid‐sparing effects of regional anesthesia, as opioids have been implicated in promoting cancer progression.^[^ ^19^ ^]^ Additionally, other studies have shown that perioperative intravenous lidocaine infusion reduces postoperative pain and opioid requirements. Lidocaine has also been demonstrated to induce apoptosis and suppress tumor growth in human breast cancer cells, as well as other cancer cell lines in vitro.^[^ ^20^ ^]^ Furthermore, it has been reported to enhance the sensitivity of breast cancer cells to chemotherapeutic agents.^[^ ^21^ ^]^ In the recent years, several studies employing lidocaine as such or embedded in either complexes or polymeric media, focusing on cancer and anesthetic therapies, were published.^[^ ^22^ ^]^ However, to the best of our knowledge, this is the first attempt of lidocaine encapsulation inside an MOF scaffold made so far, with the final use in drug delivery application. From these considerations arises the present project, which aims to utilize ZIF‐8 as a carrier for lidocaine, leveraging the pH‐sensitive properties of this MOF in the targeted and intelligent delivery of the active compound to the tumor tissue. Lidocaine has a very short half‐life,^[^ ^23^ ^]^ and controlled release from ZIF‐8 could address the limitations associated with the drug's dosing schedule. The objective of this study is to exploit our previous experience in drug encapsulation with ZIF‐8,^[^ ^24^ ^]^ by transferring such efficient and reproducible strategy for lidocaine loading inside the pores of ZIF‐8, followed by a thorough physicochemical characterization of both empty and loaded material. Once proved that we successfully encapsulated lidocaine within the pores of the MOF structure, we focused on in vitro testing in order to evaluate the effectiveness of the promising lidocaine@ZIF‐8 system for the treatment of breast cancer disease.

## 2 Experimental Section

### 2.1 Materials

Zinc nitrate hexahydrate (98%), 2‐methylimidazole (MeIm, 99%), methanol (99.8%), acetonitrile (>99.9%), hexane (>99%), acetic acid (99.7%), and phosphate‐buffered saline tablets (PBS, 0.0027M KCl; 0.137M NaCl; pH = 7.4) were purchased from Sigma‐Aldrich. Lidocaine hydrochloride (>95%) was acquired from BLD Pharma. Double deionized water was obtained from a Milli‐Q filtration/purification system (Millipore, Bedford, MA, USA). Dulbecco's modified minimum essential medium (DMEM), fetal bovine serum, penicillin, streptomycin, and trypsin were acquired from GIBCO (Uxbridge, UK). Sterile plasticware was bought from Falcon Becton‐Dickinson (Cowley, Oxford, UK), Costar (Cambridge, MA, USA) or Euroclone (Milan, Italy). The 3‐(4,5‐dimethylthiazol‐2‐yl)‐2,5‐diphenyltetrazolium (MTT) bromide reduction assay and the dimethyl sulfoxide (DMSO) were purchased by Sigma‐Aldrich Co. (St. Louis, MO, USA)

### 2.2 ZIF‐8 Synthesis

**Figure 1:**
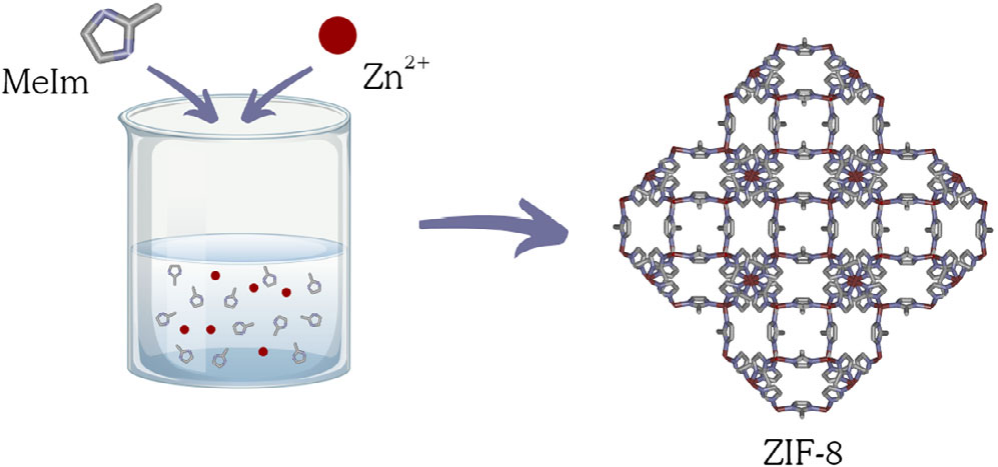
Schematic representation of the hydrothermal synthesis of ZIF‐8. The MOF structure has been obtained using iRASPA software.^[^ ^40^ ^]^ Atoms color code: Zn (red), C (gray), and N (violet).

We adapted the synthetic procedure based on the method developed by Koji Kida and coworkers.^[^ ^25^ ^]^ Briefly, 0.372 g of zinc nitrate hexahydrate was dissolved in 10 mL of deionized water and added to a solution containing 6.15 g of MeIm in 40 mL of Milli‐Q water (**Figure** **1**). The mixture, which quickly turns milky, was stirred at room temperature for 2 h. After the reaction, the resulting suspension was centrifuged at 5000 RPM for 30 min and washed three times with methanol. The products were then dried overnight under reduced pressure at 40 °C.

### 2.3 Characterization techniques

#### 2.3.1 X‐Ray Powder Diffraction (XRPD)

Gently, ground powder of ZIF‐8 and lidocaine@ZIF‐8 was deposited in the 2 mm deep hollow of a zero background plate (a properly misoriented quartz monocrystal). The diffraction experiment was performed using Cu‐Kα radiation (λ = 1.5418 Å) on a vertical‐scan Bruker AXS D8 Advance diffractometer in *θ*:*θ* mode, equipped with a Goebel Mirror and a Bruker Lynxeye linear position sensitive detector, with the following optics: primary and secondary Soller slits, 2.3° and 2.5°, respectively; divergence slit, 0.1°; receiving slit, 2.82°; and generator setting: 40 kV, 40 mA. The nominal resolution for the present set‐up is 0.08° 2*θ* (FWHM of the α 1 component) for the LaB_6_ peak at about 21.3° (2*θ*). The accurate diffraction pattern at RT of the sample was acquired in the 3°–110° 2*θ* range, with Δ2*θ* = 0.02° and exposure time 10 s/step. A Le Bail refinement was carried out in order to check the phase purity within the TOPAS‐Academic 6 software.

#### 2.3.2 Attenuated Total Reflectance‐Mid Infrared Spectroscopy (ATR‐MIR)

ATR‐MIR measurements were performed with a Perkin‐Elmer Spectrum Two instrument equipped with an atmospheric UATR Two accessory and a DTGS detector. Each spectrum was obtained by averaging eight scans with a 4 cm^−1^ resolution in the range 4000–400 cm^−1^.

#### 2.3.3 Surface Area Analysis

Surface area measurements were performed with the use of an Anton Paar Nova 800 instrument. The sample was inserted inside a calibrated volume sample holder and treated at 150 °C in dynamic vacuum for 18 h directly on the instrument for activation. N_2_ adsorption–desorption isotherms at 77 K were later on measured and specific surface area was calculated applying the brunauer‐emmett‐teller (BET) method.^[^ ^26^ ^]^

#### 2.3.4 Thermogravimetric Analysis (TGA)

TGA was carried out on a Perkin‐Elmer TGA 7 Thermogravimetric Analyzer. 1.115 mg of sample was placed on an alumina pan and heated under air. The heating ramp used was 10 K min^−1^, from 303 to 1173 K.

#### 2.3.5 Scanning Electron Microscopy (SEM)

The morphology of the samples was analyzed using SEM with a FESEM ZEISS Gemini500 instrument. Images were captured at 10 and 50 k magnifications using backscattered electrons, operating at an accelerating voltage of 5 kV. The samples were mounted on SEM stubs covered with carbon tape and subsequently coated with a thin chromium layer to increase conductivity and prevent electrical charging during imaging.

#### 2.3.6 UV–Vis Spectroscopy

UV–Vis measurements were conducted to indirectly assess the loading of the active molecule by determining its residual concentration in the synthesis solvent. This analysis was carried out using an ONDA UV‐30 SCAN spectrophotometer in the 200–300 nm range. The residual concentration of lidocaine was quantified based on the intensity of its characteristic absorption band centered at 207 nm.

### 2.4 Lidocaine Loading

Lidocaine hydrochloride is a powder that is soluble in both water and most organic solvents. Since drug loading into a porous structure is favored when there is low affinity between solvent and solute, an updated loading method was devised to overcome the challenge of solubility: stirring at low temperature (0 °C). 0.100 g of ZIF‐8 and 0.150 g of lidocaine hydrochloride were dissolved in 100 mL of solvent (methanol, acetonitrile, or hexane). The mixture was sonicated for 5 min to allow ZIF‐8 to homogeneously disperse in the liquid phase, placed in a beaker cooled to 0 °C using a chiller, and finally stirred for 24 h. After the reaction time had elapsed, the resulting suspension was centrifuged at 5000 RPM for 30 min and washed twice with methanol. The product was then dried overnight under reduced pressure at 40 °C.

### 2.5 Release Test

Lidocaine release tests were conducted by dispersing 100 mg of the previously synthesized Lidocaine@ZIF‐8 system in a solution of 9 mL acetate buffer (pH = 5.5) and 1 mL MeOH. The same set‐up was used with PBS (pH = 7.4). The suspension was kept under gentle agitation at 37 °C, simulating average body temperature, for several days.

### 2.6 MTT Assay

Metabolic activity of MDA‐MB‐231 breast cancer cells was assessed by the 3‐(4,5‐dimethylthiazol‐2‐yl)‐2,5‐diphenyltetrazolium (MTT) bromide reduction assay. Lidocaine, ZIF‐8, or lidocaine@ZIF‐8, were dissolved in DMEM + 1% Pen/Strep and 1% L‐Glut (cell culture vehicle). Tumor cells (9000 cells/cm^2^) were seeded in 96‐well plates and then treated with vehicle, lidocaine 0.01 mg mL^−1^, ZIF‐8 0.01 mg mL^−1^, or lidocaine@ZIF‐8 0.01 mg mL^−1^, alternatively. MTT was dissolved in dulbecco's phosphate‐buffered saline at 5 mg ml^−1^ concentration and added at 1:6_v/v_ ratio directly into the cell supernatant. Three hours later, medium was removed and DMSO was added to dissolve the precipitated formazan salts arising from the reaction. Plates were shaken at 320 RPM on an orbital shaker for 10 min, then absorbance at 595 nm was recorded and plotted as X‐fold to the absorbance of the vehicle.

## 3 Results and Discussion

### 3.1 Synthesis and Characterization

ZIF‐8 is one of the most widely used and explored MOFs in the biomedical field, and currently, there are numerous synthetic methods to obtain ZIF‐8, even with slight structural variations.^[^ ^27^ ^]^ In this work, ZIF‐8 was synthesized, loaded, and characterized according to an optimized method that we developed in a previous study.^[^ ^24^ ^]^ This method also involves hydrothermal synthesis, which is more suitable for biomedical applications since water is used as the solvent, allowing for the production of particles maintaining their properties intact.

**Figure 2:**
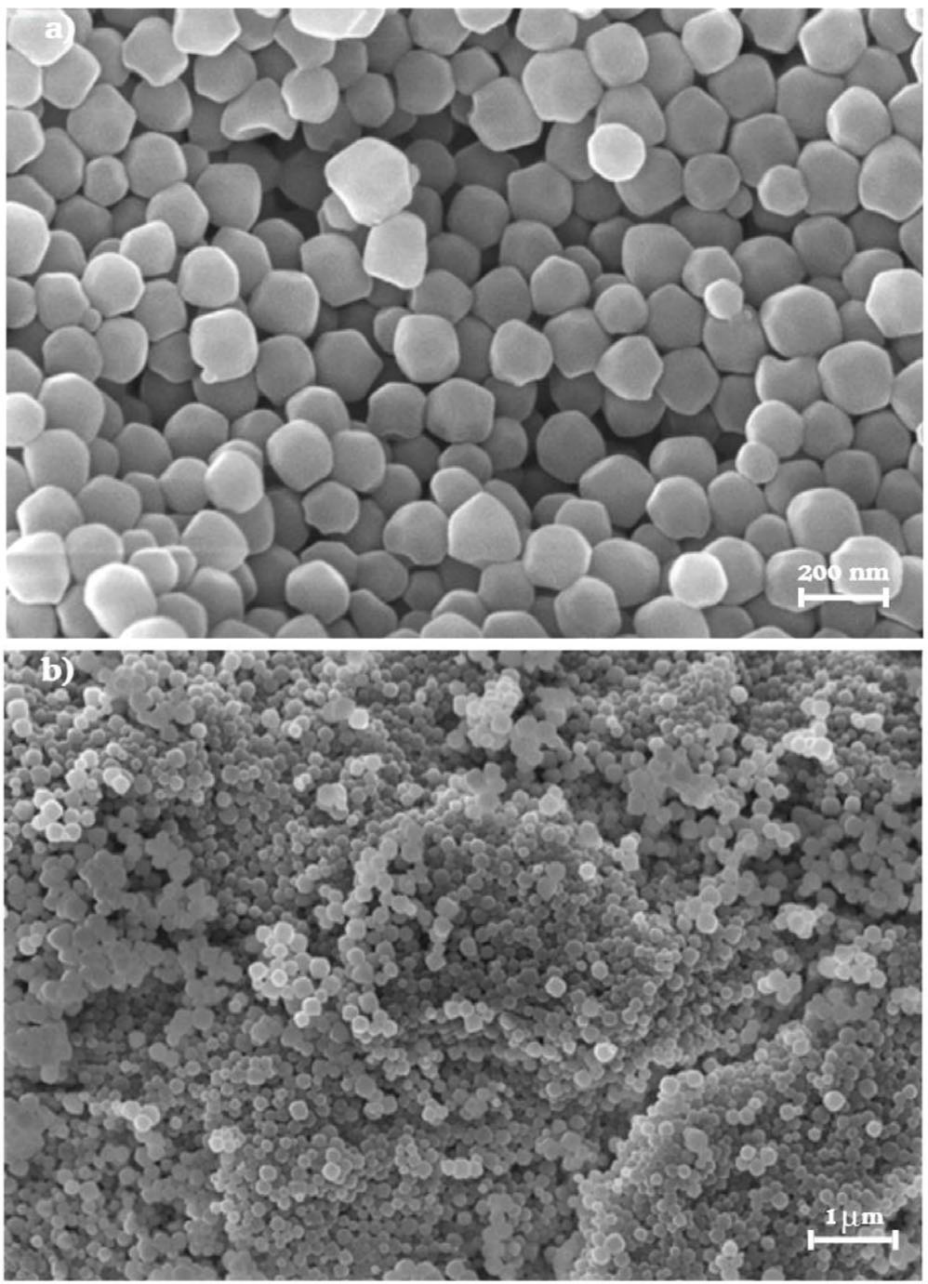
SEM images collected at a) 50 kX and b) 10 kX of as‐prepared ZIF‐8.

This method yields ZIF‐8 crystals with a size of ≈150 nm and a homogeneous morphology, as shown in **Figure** **2**. BET surface area (1813 m^2^ g^−1^), measured by nitrogen adsorption at 77 K, confirms that the MOF morphological properties are comparable to those already reported in the literature (Figure S1a, Supporting Information).^[^ ^28^ ^]^

Once ZIF‐8 was obtained, the next step was optimizing the loading of lidocaine into the crystal cavities. This active principle is in the form of a chloride salt and is highly soluble in most organic solvents, making the loading process challenging due to its affinity with the solvent. To overcome this obstacle, the loading procedure was carried out in a thermostatic beaker (at T = 0 °C), in order to reduce lidocaine's solubility. The experiments were conducted using a ZIF‐8:lidocaine weight ratio of 2:3, with the mixture stirred for 24 h. To identify the best solvent for loading, the tests were performed varying the polarity of the medium, without compromising ZIF‐8 suspendability: the selected solvents in which the loading procedure was attempted were methanol, acetonitrile, and hexane, respectively. The loading efficiency was determined by calculating the ratio between the initial and final concentrations of lidocaine present in both the synthesis and washing solutions, exploiting the absorption of lidocaine centered at 215 nm by means of UV–Vis spectroscopy. As expected from the UV–Vis spectra (Figure S2, Supporting Information),^[^ ^29^ ^]^ methanol proved to be the least effective solvent for drug loading into ZIF‐8, as the lidocaine concentration in the wash solutions remained equal to the initial value. A slight improvement was observed with acetonitrile as the solvent, yielding an efficiency of 6.5%. However, the highest efficiency, ≈14%_wt_., was achieved using hexane, in which lidocaine has the lowest solubility among the selected solvents, thus facilitating its interaction with the MOF pores. Despite the mass loading might seem low, it approximately corresponds to the occupancy of one lidocaine molecule per pore of ZIF‐8, indicating a good overall loading process. Indeed, previously reported studies^[22b]^ indicate a loading of drug perfectly comparable to the one presented in this work, while others present a loading level definitely lower.^[22c]^

**Figure 3:**
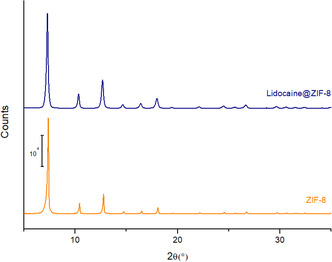
XRPD patterns of ZIF‐8 (orange) and lidocaine@ZIF‐8 (blue).

**Figure 4:**
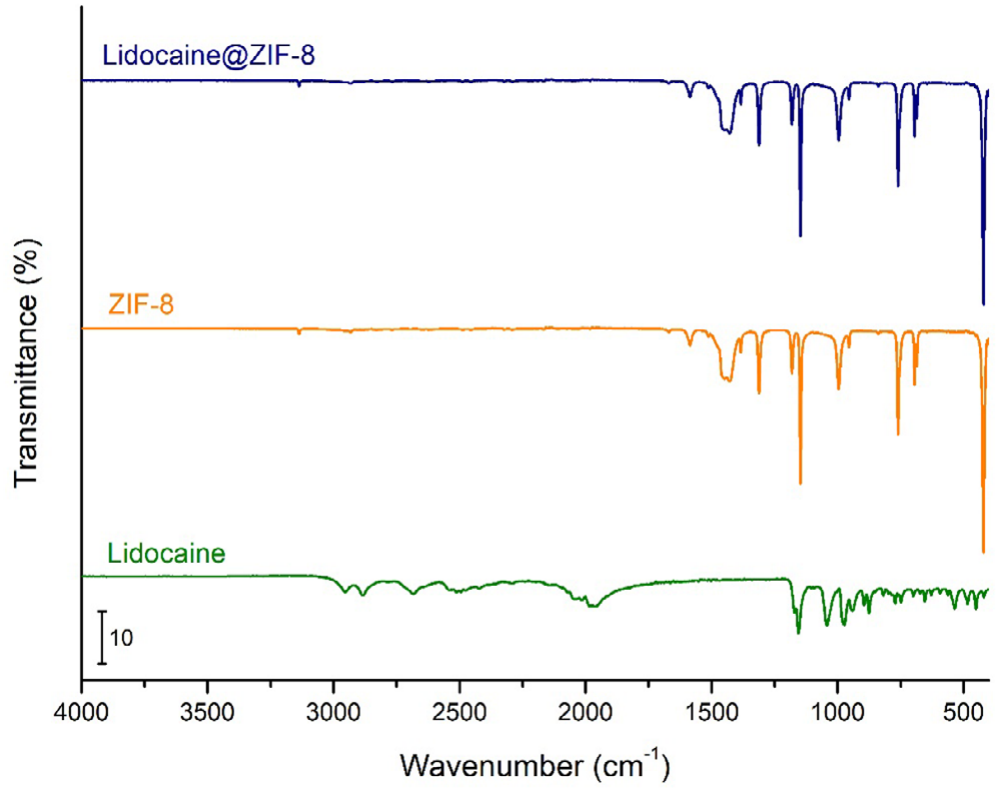
ATR‐MIR spectra of pure lidocaine (green), ZIF‐8 (orange), and lidocaine@ZIF‐8 (blue).

After obtaining the lidocaine‐loaded ZIF‐8 system (lidocaine@ZIF‐8), the material was characterized to detect any differences from pure ZIF‐8 and determining whether lidocaine was encapsulated within the pores or merely adsorbed onto the crystal surface. Initially, XRPD analysis was performed to assess any possible loss of crystallinity in the MOF. As shown in **Figure** **3**, the crystalline patterns are perfectly overlaid, indicating no degradation of ZIF‐8 and excluding the appearance of new peaks associated with lidocaine crystals or eventual additional phases.^[^ ^30^ ^]^ This result is further confirmed by SEM images (Figure S3, Supporting Information), which show crystals with the same size and morphology as the parent material. Additionally, ATR‐MIR spectroscopy was used to confirm the location of the lidocaine. If the drug was adsorbed on the surface, characteristic peaks of its functional groups would be visible (see **Figure** **4**). However, the spectra of pure ZIF‐8 and lidocaine@ZIF‐8 are perfectly overlaid, confirming successful encapsulation of lidocaine within the pores. This because it has been reported that biological active molecules confined inside small cages of materials provoke a strong decrease of the overall IR intensity of the encapsulated species.^[^ ^24^, ^31^ ^]^ In addition, once the material was obtained, the surface area was measured again by nitrogen physisorption at 77 K to assess any differences in the inner surface area values. Indeed, it can be clearly observed that there is a slight decrease in the surface area value after loading (passing from 1813 to 1482 m^2^ g^−1^), thus indicating the presence of drug within the pores of the MOF (Figure S1b, Supporting Information).

Finally, to determine the composition of the lidocaine@ZIF‐8 system and to validate the findings obtained from UV–Vis spectroscopy, TGA was performed on both drug‐loaded and bare ZIF‐8. As shown in Figure S4, Supporting Information, unloaded ZIF‐8 displays the typical mass loss for such systems (around 350 °C), leaving behind ≈36%_wt_. of ZnO coming from MOF degradation, corresponding to the mass remaining for a perfectly stoichiometric ZIF‐8 material.^[^ ^6^, ^32^ ^]^ The curve of the drug‐loaded material differs from the previous one primarily in the slight initial weight loss around 100 °C, which corresponds to the water adsorbed by lidocaine@ZIF‐8 sample (≈2%). This is due to better interaction of lidocaine with water from moisture with respect to the bare MOF. A further and more significant weight loss at 150 °C is associated to the presence of lidocaine, accounting for 14%_wt_. of the newly formed material. The temperature associated to this weight loss is in good agreement with lidocaine boiling temperature,^[^ ^33^ ^]^ meaning that it has been released from the pores of the material.

### 3.2 Release Test

**Figure 5:**
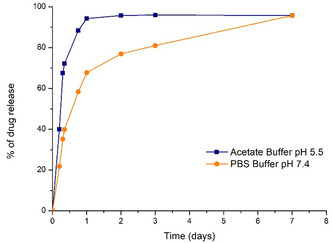
Drug release as a function of time obtained from UV–Vis data of the dialysis solution at pH = 5.5 and pH = 7.4, respectively.

There are several works in the literature demonstrating the stability and pH‐sensitive properties of ZIF‐8.^[^ ^34^ ^]^ Therefore, similarly to previous studies,^[^ ^24^ ^]^ the final aim of this preliminary study is to observe the release behavior of lidocaine@ZIF‐8 under different pH conditions. The graphs report the percentage of drug released, based on the absorbance values ratio (obtained through UV–Vis spectroscopy) between the release solution and the one coming from the theoretical amount loaded into the MOF. These data clearly illustrate the different behaviors of lidocaine release depending on the pH of the working solution (**Figure** **5**). Indeed, in an acidic environment (pH = 5.5), 94% of the drug is released within 24 h; conversely, in physiological conditions (pH = 7.4), only 67% of the encapsulated lidocaine is released in the same period. Under acidic conditions, the release rapidly reaches a plateau after 36 h, achieving a total drug delivery of 95.7%. In contrast, to reach the same level of release under neutral pH, a period of 7 days is required. These results suggest a controlled drug release depending on the pH level in the microenvironment. In the case of breast cancer, a more rapid release of lidocaine would occur in the acidic tumoral regions; however, in early‐stage treatment, this mechanism could be advantageous by accelerating the release of the active molecule in response to localized acidification in tumor tissues.

### 3.3 In vitro Evaluation of Lidocaine@ZIF‐8 Effect on Breast Cancer Cells Viability

**Figure 6:**
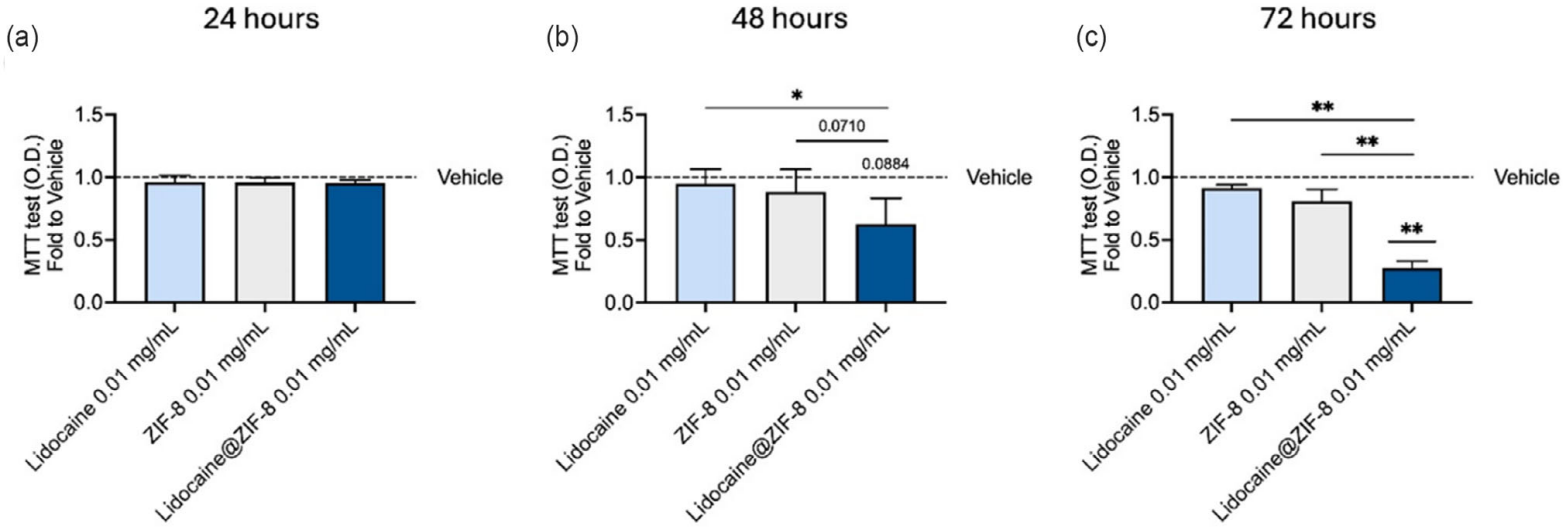
*Effect of lidocaine@ZIF‐8 on MDA‐MB‐231 breast cancer cells viability:* MDA‐MB‐231 have been plated at a density of 9000 cells cm^−2^ and, the day after, treated for a) 24, b) 48, and c) 72 h, with vehicle, lidocaine 0.01 mg mL^−1^, ZIF‐8 0.01 mg mL^−1^, and lidocaine@ZIF‐8 0.01 mg mL^−1^, respectively. Cells viability was assessed by MTT assay (Number of independent experiment (N) = 3; *p‐value (p)<0.05, **p < 0.01 versus Lidocaine@ZIF‐8 0.01 mg mL^−1^; paired *t*‐test; dot line  =  vehicle set at 1).

In recent years, it has been observed that lidocaine induces metabolomic changes in breast cancer cells profile,^[^ ^35^ ^]^ and apoptosis through VDAC1 expression.^[^ ^36^ ^]^ Moreover, it has been reported that lidocaine can suppress cancer cell growth via blocking voltage‐gated sodium channels.^[^ ^37^ ^]^ Although this molecule shows promise in the treatment of breast cancers, to date it is not yet possible to define absolutely what the exact mechanism of action is. Therefore, we finally investigated the effect of lidocaine@ZIF‐8 on MDA‐MB‐231 breast cancer cells viability. After a short period of 24 h, treatment of breast cancer cells with lidocaine@ZIF‐8 0.01 mg mL^−1^ did not show any significant differences when compared to lidocaine 0.01 mg mL^−1^, with ZIF‐8 0.01 mg mL^−1^, or vehicle, respectively (**Figure** **6**a). Hence, we next treated MDA‐MB‐231 cells with lidocaine@ZIF‐8 0.01 mg mL^−1^ for a period of 48 h. Intriguingly, the treatment showed a *trend* to decrease cells viability when compared to vehicle (*p*‐value = 0.0884) and ZIF‐8 0.01 mg mL^−1^ (*p*‐value = 0.0710), whereas a significant decrease was observed when compared to lidocaine 0.01 mg mL^−1^ (Figure 6b). To deeply characterize the time‐dependent effect of lidocaine@ZIF‐8 on MDA‐MB‐231 cells, we performed a last treatment of 72 h. Interestingly, lidocaine@ZIF‐8 treatment of breast cancer cells showed a significant reduction of cells viability when compared to lidocaine 0.01 mg /mL^−1^, with ZIF‐8 0.01 mg mL^−1^, and vehicle, respectively (Figure 6c).

These results may suggest a time‐dependent drug release, possibly correlated with the pH level in the tumor microenvironment, corroborating data collected from the release tests previously described. One noticeable fact is the difference in time response between the effect of lidocanine@ZIF‐8 observed on cancer cells and the pH‐dependent drug release curves reported in Figure 5. Indeed, at pH = 5.5, the total release of lidocaine was observed already at 36 h. Nevertheless, a noticeable effect on the cells inoculated with lidocaine@ZIF‐8 started to be visible only after 48 h, reaching its maximum at 72 h. Such delay might be ascribable to differences in pH values of the cell environment with respect to the one of the test release solution: even slight differences in acidity of the medium has been reported to greatly affect ZIF‐8 decomposition,^[^ ^38^ ^]^ thus delaying the drug release. A possible explanation is that we cannot measure pH variation of the working environment, which is strongly influenced by the number of cancer cell present, impacting on the acidity of their surroundings.

However, providing that these are only preliminary results, further studies are required to evaluate the efficacy of the lidocaine@ZIF‐8 system and to assess the cytotoxicity of the pure and loaded MOF.

## 4 Conclusions

The aim of this study was to investigate the encapsulation of lidocaine within the cavities of ZIF‐8, an MOF highly sensitive to pH variations. This pH‐responsive property enables ZIF‐8 to release drugs in a controlled manner in the tumor microenvironment. Recent research has shown that, in addition to being a commonly used local anesthetic, lidocaine possesses the ability to inhibit the metastatic potential of breast cancer cells,^[^ ^36^, ^39^ ^]^ one of the most widespread cancers overall, preventing them from invading other tissues. After synthesizing ZIF‐8 using a well‐established synthetic strategy, an alternative method to conventional stirring was employed to encapsulate lidocaine. This approach involved lowering the working temperature to reduce the solubility of the drug. Once the lidocaine@ZIF‐8 system was obtained, it was extensively characterized using various techniques and subsequently tested on cancer cell cultures to evaluate its efficacy. Notably, ZIF‐8 facilitated lidocaine release, reducing breast cancer cells viability in a time and pH‐dependent manner. Interestingly, despite the low amount of loaded lidocaine (only 14 %_wt_.) its effect was much higher with respect to the same amount of free drug. Indeed, a higher loading, implying multiple cage occupancy by the drug molecules, could have resulted in a slower molecular diffusion or drug release in unnecessary areas, thus potentially reducing the efficacy of the system as a whole. This result is extremely important, since it aims to reduce the dosage of drugs in order to minimize accumulation, excretion, and possible side effects. However, to fully understand the mechanism underlying the action of lidocaine for the treatment of breast cancer, more complex in vitro studies are needed in the future before moving on to in vivo studies.

## Conflict of Interest

The authors declare no conflict of interest.

## Supporting information

Supplementary Material

## Data Availability

The data that support the findings of this study are available from the corresponding author upon reasonable request.
