# Supplementary material for: Targeted Delivery of Lidocaine in Breast Cancer Cells via Zeolitic Imidazolate Framework‐8 Nanoparticles
Source: Chemphyschem. 2025 Jun 24;26(16):e202401128. doi: 10.1002/cphc.202401128 (PMC12388163; doi:10.1002/cphc.202401128)
Supplement: Supplementary file 1 — Supplementary Material [file CPHC-26-e202401128-s001.pdf]

SUPPORTING INFORMATION

Targeted Delivery of Lidocaine in Breast Cancer Cells *via* ZIF-8 Nanoparticles

Nicola di Nicola[a,b], Luca Giacchi[c], Marco Vandone[d], Marcello Crucianelli[a,b], Leonardo Guidoni[a], Valentina Colombo[d], Nadia Rucci[c], Andrea Lazzarini[a,b,*]

[a] Department of Physical and Chemical Sciences (DSFC), University of L’Aquila, Via Vetoio (“A.C. De Meis” building), L'Aquila, 67100, Italy.

[b] UdR INSTM of L’Aquila, University of L’Aquila, Via Vetoio (“A.C. De Meis” building), L'Aquila, 67100, Italy.

[c] Department of Biotechnological and Applied Clinical Sciences (DISCAB), University of L’Aquila, Via Vetoio (“A.C. De Meis” building), L'Aquila, 67100, Italy.

[d] Department of Chemistry and UdR INSTM of Milano, University of Milano, Via C. Golgi 19, Milano, 20133, Italy.

[*] Corresponding author: andrea.lazzarini@univaq.it

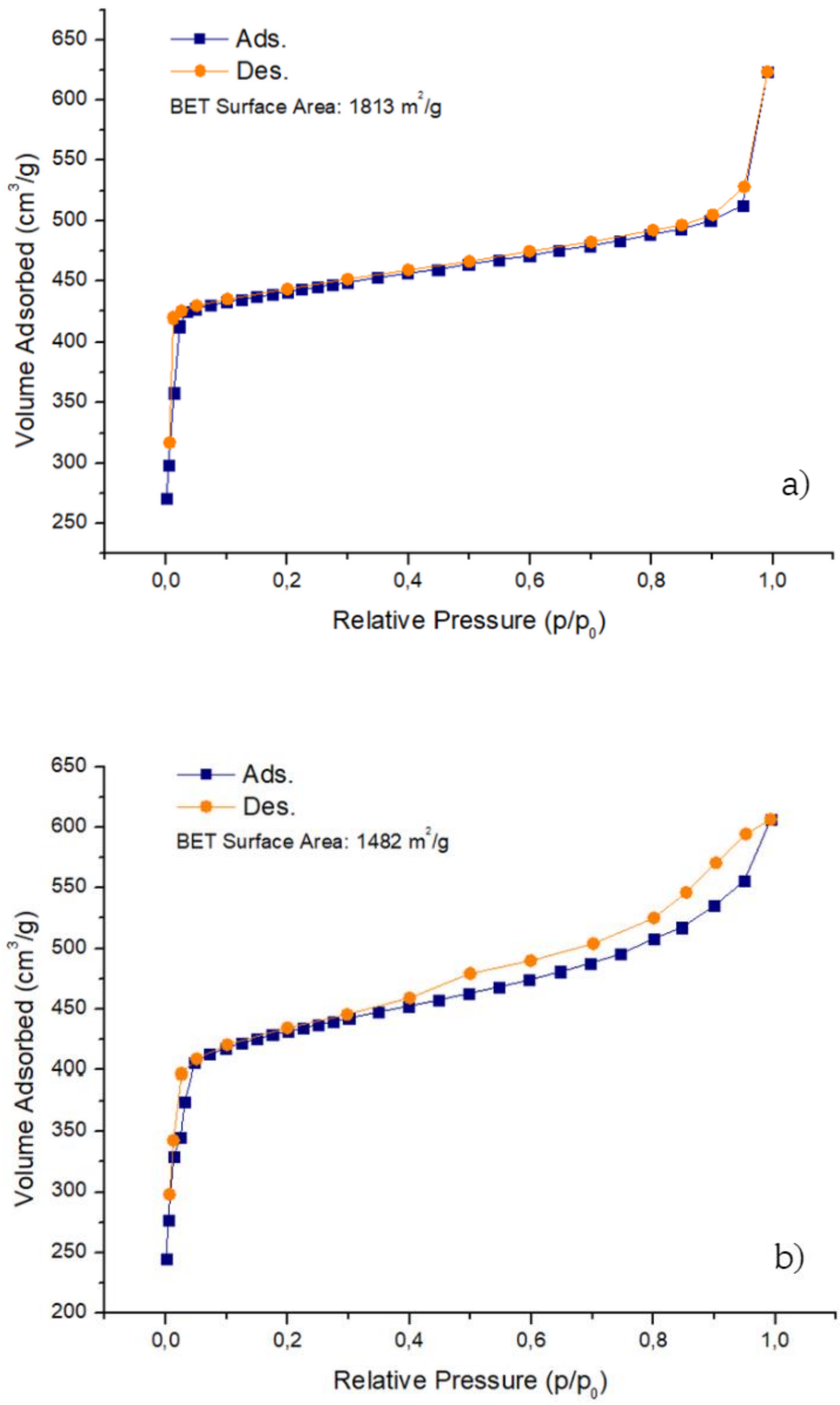

Figure S1. N_2 adsorption-desorption isotherms at 77 K of ZIF-8 (a) and lidocaine@ZIF-8 (b).

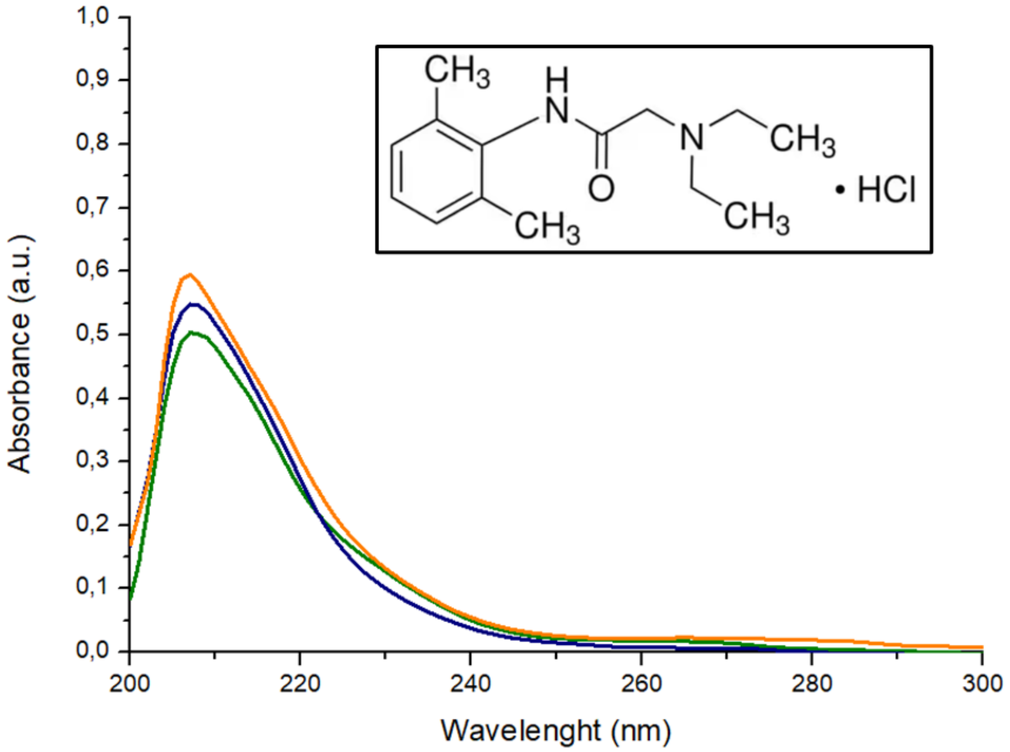

Figure S2. UV-Vis spectra of synthesis and washing solutions (i.e. hexane in green, methanol in orange, and acetonitrile in blue) after drug loading for the different solvents employed. Inset reports the scheme of the lidocaine hydrochloride molecule.

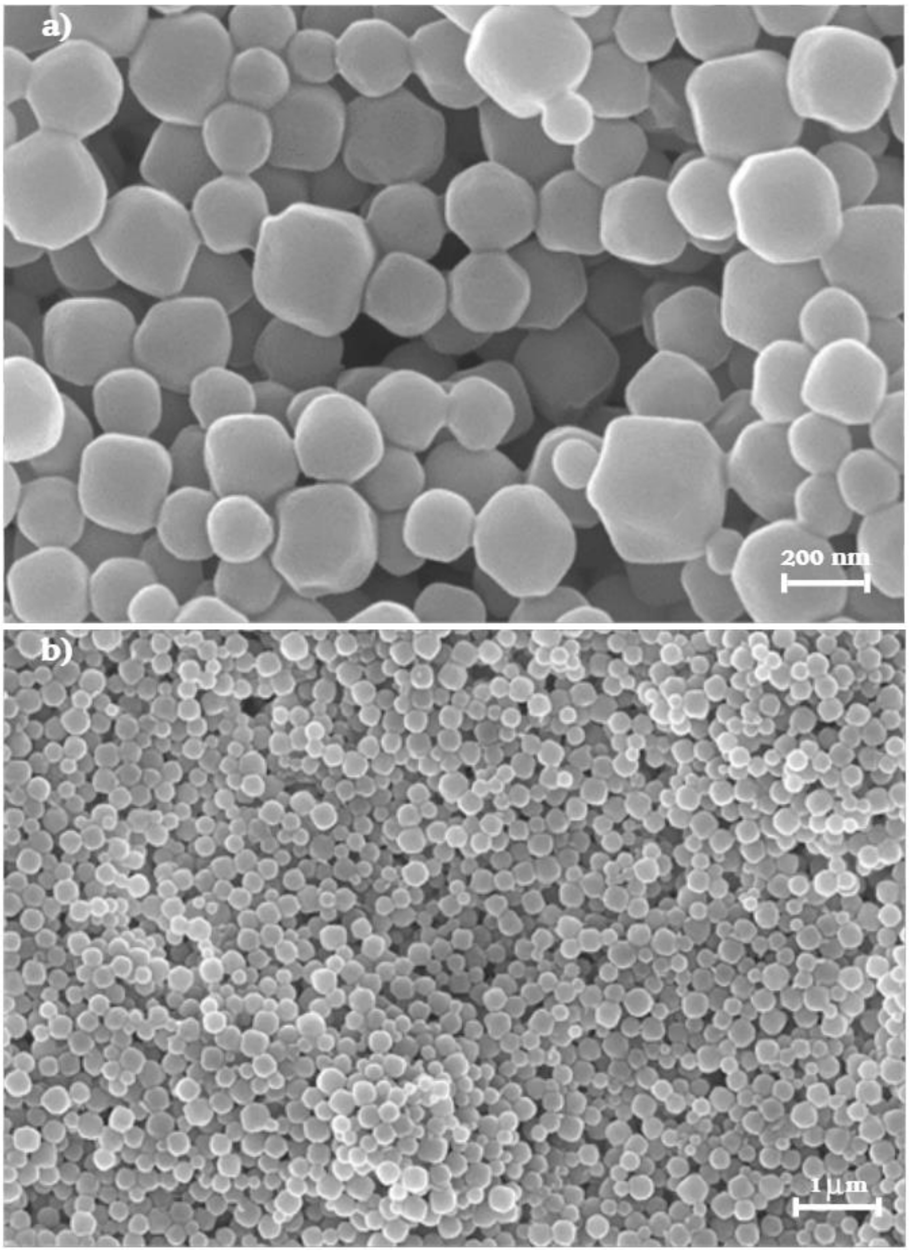

Figure S3. SEM images collected at 50 kX (a) and 10 kX (b) of lidocaine@ZIF-8.

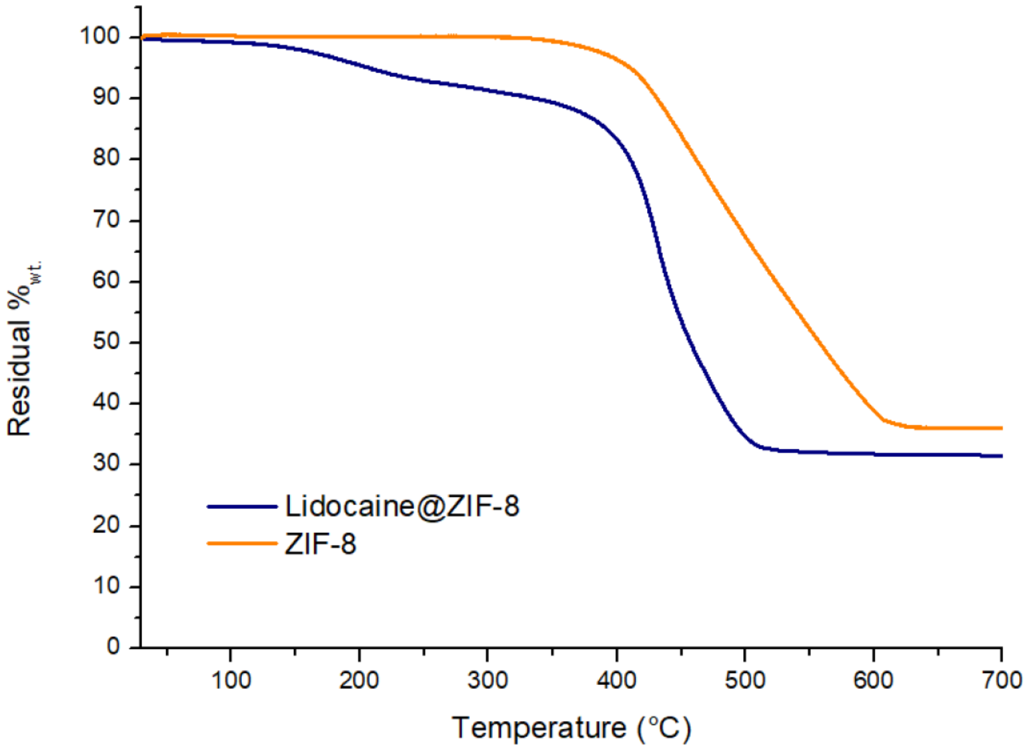

Figure S4. TGA curves of ZIF-8 (orange) and lidocaine@ZIF-8 (blue).
